# Impact of a Multidimensional Community-Based Intervention on the Feeling of Unwanted Loneliness and Its Consequences: A Quasi-Experimental Study

**DOI:** 10.3390/healthcare13121465

**Published:** 2025-06-18

**Authors:** Alba Francisco-Sánchez, Sofía Martínez-León, Alejandro García-Pérez, Juan Andrés Báez-Hernández, Martín Rodríguez-Álvaro, Alfonso Miguel García-Hernández

**Affiliations:** 1 Primary Health Care, La Palma Health Services, The Canary Islands Health Service, 38713 Breña Alta, Spain; afrasanb@gobiernodecanarias.org (A.F.-S.); agarperp@gobiernodecanarias.org (A.G.-P.); jbaeher@gobiernodecanarias.org (J.A.B.-H.); 2 Nursing Department, Healthcare Sciences School, University of La Laguna, 38200 Santa Cruz de Tenerife, Spain; almigar@ull.edu.es

**Keywords:** loneliness, social support, community health nursing, health education

## Abstract

**Background/Objectives**: Unwanted loneliness is the gap between the social relations a person has and those they want. The main objective of this research is to assess the impact of a multidimensional community-based intervention on the feeling of unwanted loneliness in the population over the age of 65 years old who live alone, are under social risk, or are socially isolated living on La Palma island. **Methods**: A quasi-experimental study was designed with pre- and post-intervention (at three months) measurements, with no control group or randomization. A sample comprising 90 subjects was estimated for a small–moderate (0.3) or large (0.8) effect size, with a significance level (α) of 0.05 and a power (1 − β) of 0.8. **Results**: The intervention was initiated with 90 participants in 8 of the 9 Basic Health Districts from the La Palma Health Area. A moderate effect size (d = −0.77; 95%CI [−1.02, −0.52]) was evidenced in self-perceived loneliness. Three months after the proposed community-based intervention, significant differences were evidenced in adequate eating habits, physical activity, support network, anxiety, depression, and perceived social support. **Conclusions**: Compartiendo Salud (Sharing Health) presents promising results, as it exerts positive effects on health management among older adults that live alone. The results of this intervention could serve as a model to design replicable strategies in other communities, improving the quality of life and levels of perceived social support in older adults.

## 1. Introduction

Approximately four out of every ten older adults live in single-person households [1]. Loneliness affects a significant proportion of older adults across Europe. The prevalence of loneliness in this population varies widely, ranging from 3% in Denmark to 35% in Moldova [2]. In Spain, the prevalence of unwanted loneliness in older adults ranges between 14% and 16.3% [3]. This condition represents a growing public health concern due its extensive consequences.

Notably, the annual cost associated with unwanted loneliness in Spain has been estimated at EUR 14,000,000, including both tangible and intangible costs, as well as those associated with productivity losses. Beyond its financial impact, unwanted loneliness constitutes a public health issue that demands an intersectoral response, involving collaborative efforts among the person, the family, the community, the social sectors, the public health authorities, and Primary Health Care.

Loneliness is strongly associated with adverse health outcomes, including mental health disorders, and may act as an independent risk factor for depression. Moreover, it is associated with adverse health outcomes such as elevated blood pressure, poorer sleep, immune responses to stress, and poorer cognition over time in older adults [4]. The absence of personal connections is related to the adoption of worse life habits and to increased morbidity, with the consequent higher risk of suffering cardiovascular and endocrine diseases, mental health pathologies, and falls.

The influence of social relationships on the risk of death is comparable to that of well-established mortality risk factors such as smoking and alcohol consumption, and exceeds the influence of other risk factors, such as physical inactivity and obesity [5]. Studies have shown that the risk of premature mortality increases by 32% among people living alone, by 26% in those who feel lonely, and by 9% among the socially isolated [6]. Sedentary lifestyles, smoking, alcohol consumption, worse eating habits, sleep quality and quantity, poor adherence to treatments, and higher resource consumption are some problems associated with loneliness [7,8,9,10].

Particularly worrying is the feeling of unwanted loneliness. Unwanted loneliness is defined as the discrepancy between an individual’s existing social relationships and those they desire. If this gap is ample or persistent, it produces pain, fear, anguish, or sadness. Therefore, unwanted loneliness is a negative personal experience in which a person has the need to communicate with others and perceives deficits in their social relations either because they socialize less than they would like to or because the relationships they have fail to offer them the emotional support they want. This feeling can emerge when the social environment does not favor establishing interpersonal relationships or when these relationships are scarcely satisfactory, with the possibility of this situation affecting the person’s health [11].

In this context, we must not ignore the invisibility of older people and their needs, creating divided societies [12]. In addition to the structural factors inherent in modern society, changes in care systems and housing arrangements have contributed to increased loneliness. The increasing use of digital communication, changes in neighborhood social dynamics, and evolving housing and caregiving models promote individualism and have weakened interpersonal relationships among older adults [13]. This exerts a negative effect on older adults’ well-being and quality of life.

Public policies aimed at combating isolation through specific support and social intervention programs should facilitate social contact, making it difficult for feelings of loneliness to arise. At the national level, no specific policies have been developed to combat unwanted loneliness in a comprehensive and planned manner. However, the growing concern about the phenomenon has put unwanted loneliness on the political agenda. Thus, actions and projects are emerging in different areas: regional and provincial public administrations, local entities, and social initiatives. The Institute for the Elderly and Social Services (IMSERSO) is leading the creation of a National Strategy against Unwanted Loneliness, which is yet to come [13].

At the local, national, and international levels alike, several awareness-raising campaigns on the problem of loneliness and isolation in the community have been carried out. Different strategies have been studied, with the following standing out: interventions that offer socialization groups and include a psychosocial approach, humor therapies, physical activities and therapy with animals, and workshops on digital literacy, cooking, gardening, music, and reading, among others [14].

In our country, multiple actions have been carried out with the objective of reducing this feeling: at the national level, we can mention the Unwanted Loneliness State Observatory [13], as well as different programs deployed in autonomous communities, such as “Barcelona contra la soledad” (“Barcelona against loneliness”) [15], “I Plan Integral de Mayores de Canarias y Soledad No Deseada” (“First Comprehensive Plan for Canarian Older Adults and Unwanted Loneliness”), and, at the local level, “Enrédate” (“Get Networked”) by Red Cross La Palma [16].

Evidence suggests that interventions targeted at reducing loneliness by means of home visits or phone follow-up are rarely effective. Conversely, significant improvements are achieved by implementing activities that promote participation in community-based programs and enhance personal autonomy. The recent meta-analysis by Saun-Fun Yu et al. [17], which includes 60 studies and more than 13,000 participants, indicated the positive effects of psychological interventions, especially those that include counseling. Group exercise interventions with social commitment improve the emotional negativity associated with loneliness and enhance social connectivity. Especially in older adults that have already reported loneliness, multidimensional interventions show a positive effect; however, they seem less effective than those focused on a single domain [17]. Increasing mental vitality and self-esteem, fostering a positive perspective regarding the social context, and improving emotional coping and resilience are interventions that can enhance social connection [18].

On the other hand, as Zahaira et al. point out, “nurses have the knowledge and skills to detect health problems in their early stages, such as identifying cases of unwanted loneliness. Furthermore, due to their holistic approach, nurses are well positioned not only to select and implement the most appropriate assessment tools based on patients’ needs, but also to coordinate and lead multidisciplinary teams for interventions” [19].

Although it is a subjective and complex concept, there are different validated tools to measure unwanted loneliness. The University of California at Los Angeles (UCLA) Loneliness Scale and the De Jong Gierveld Loneliness Scale are the most used worldwide [17,20].

The main objective of this research is to evaluate the impact of a multidimensional community-based intervention on unwanted loneliness in adults over 65 years old living alone, at social risk, or socially isolated in La Palma, Canary Islands, Spain. The secondary objectives proposed are:

- Increasing the number of opportunities for social interaction during the intervention;
- Contributing tools to improve the participants’ social skills in the intervention;
- Fostering self-care and healthy habits in the participants;
- Reducing maladaptive thoughts;
- Educating on sleep hygiene measures and non-pharmacological treatments.

The null hypothesis (H0) of this research is that the multidimensional intervention will not significantly affect the feeling of unwanted loneliness and the health management of these subjects in the target population.

## 2. Materials and Methods

### 2.1. Design and Sampling Method

A quasi-experimental study was designed with pre- and post-intervention (at three months) measurements, with no control group or randomization.

The study population comprised older adults living alone that are at risk of loneliness or are socially isolated in the La Palma Health Area, Canary Islands, Spain. The accessible population consisted of individuals with a record of at least one of these three characteristics identified in their electronic medical histories at Primary Health Care (Drago-AP).

To gauge the sample according to the expected effect size, Cohen’s proposal for this size was taken as a reference [21,22]. The sample was calculated with the G*Power 3.1 software [23] for paired samples (with pre- and post-intervention measurements in the same subjects), with a significance level (α) of 0.05 and a power (1 − β) of 0.8, standard values to ensure validity of the results. For a small–moderate effect size (dz = 0.3), the estimated sample size was 71 subjects. Given the vulnerability inherent to the study population, it was increased by 30% to account for possible losses and withdrawals, resulting in a final sample comprising 90 individuals.

The following inclusion criteria were considered (record in the electronic medical record in Primary Health Care): (a) people over the age of 65 and living alone, (b) Nursing Diagnosis of social risk, or (c) Nursing Diagnosis of social isolation. In turn, these exclusion criteria were applied: (a) presenting moderate/severe cognitive decline or Pfeiffer [24] scores above 6, (b) individuals with uncorrected hearing deficits that hinder group interaction, and (c) participants not subjected to the pre- and post-interventions measurements, as well as those that did not take part in at least four of the five sessions scheduled. Given the participants’ characteristics, death of a participant or meeting any exclusion criteria during the project were considered criteria to remove them from the project.

Convenience, non-random sampling was performed, including the participants meeting the established criteria and living in the La Palma Health Area during the study period. The sample was selected by resorting to three strategies: active recruiting through phone calls to the participants that meet the inclusion criteria (list provided by the Canarian Health Service information services), opportunistic recruitment of participants that attend Primary Care consultations, and participants referred from community agents (municipal social services, Red Cross). The period of recruitment was carried out during the time frame from September 2023 to January 2024.

The study was reported according to the Transparent Reporting of Evaluations with Non-Randomized Designs (TREND) checklist for non-randomized trials [25].

### 2.2. Study Setting

The La Palma Health Area is one of seven healthcare areas in the Canary Islands Health Service (SCS), which is part of Spain’s National Health System and delivers public healthcare services to the population of the Canary Islands. This health area serves an attached population consisting of 78,664 individuals. There are 9 Basic Health Districts. In Primary Health, nursing care is provided by approximately 122 nurses. The percentage of the population aged over 65 years old in La Palma is 21.19%, which corresponds to 17,638 inhabitants. In September 2023, and according to the health history records, 464 non-confined participants over the age of 65 from the La Palma Health Area had a record of any of the three inclusion criteria. In Primary Health, the La Palma Health Area includes the following care centers [26]:

- Santa Cruz de La Palma Basic Health District (Santa Cruz de La Palma Health Centre, Puntallana Local Medical Office);
- Las Breñas Basic Health District (Breña Alta Health Centre, Breña Baja Local Medical Office);
- Mazo Basic Health District (Mazo Health Centre, Fuencaliente Local Medical Office);
- El Paso Basic Health District (El Paso Health Centre, Las Manchas Local Medical Office);
- Los Llanos de Aridane Basic Health District (Llanos de Aridane Health Centre);
- Tazacorte Basic Health District (Tazacorte Health Centre, Puerto Tazacorte Local Medical Office);
- Tijarafe Basic Health District (Tijarafe Health Centre, Puntagorda Local Medical Office);
- Garafía Basic Health District (Garafía Health Centre, Los Franceses Local Medical Office);
- San Andrés y Sauces Basic Health District (San Andrés Health Centre, Barlovento Local Medical Office, Gallegos Local Medical Office).

Primary Health Care works with Drago-AP electronic medical histories. In all, 99% of the individuals from the attached population had a record in their electronic medical history. The Drago-AP tool operates on an ORACLE database. Community nurses have access to a specific module to log the care they provide. It includes a specific model to record and plan care based on a structured assessment by health standards, which gives rise to defining care needs identified with the Nursing Diagnoses (NDs) from the NANDA-I classification [27]. The nurse creates a care plan by starting with an assessment based on Marjory Gordon’s health patterns (HPs) [28]. The record confirming that a person lives alone is made by assessing health pattern number 8 (role/relationship). When the research was conducted, this health history was combined with the 12th edition NANDA-I Nursing Diagnoses classification, which includes the risk of loneliness and social isolation Nursing Diagnosis labels.

### 2.3. Nursing Intervention Design and Preparation

The intervention proposed (*Compartiendo Salud*) is a multicomponent intervention based on prevention and on promoting the health of older adults that live alone. It was designed by a multidisciplinary team headed by nurse A.F.S., a specialist in family and community nursing. Her design includes nurses, physicians, physical therapists, a pharmacist, and a psychologist.

To ensure fidelity and a coherent application of the intervention, the research team provided approximately 10 h of training to 43 health professionals that were interested in taking part in the project. Nevertheless, the presence of at least one team member was guaranteed in all the interventions at each of the basic health districts. Likewise, all Primary Care health professionals from La Palma had direct access to a shared folder with the content of all workshops and the audiovisual materials required to develop them.

The community-based intervention consists of five workshops that are carried out during a week (one day for each workshop). The sessions (which last 2.5 h each) are designed in an expository and participatory way, using gaming dynamics that foster learning and social interaction. The content of the workshops is the same for all BHDs. Each workshop addresses risk factors that predispose the population to experiencing more unwanted loneliness and social isolation. The concepts and dynamics corresponding to each of the five sessions that comprise the intervention are presented in Table 1.

After devising the intervention and training the professionals, we proceeded to schedule the dates with the Primary Care teams from the different BHDs, City Halls, and Primary Care Management Offices. Finally, a total of 8 groups were formed, one for each BHD, except for Garafía. The intervention, including the reassessment of participants, was carried out during the time frame from October 2023 to April 2024. Specifically, *Compartiendo Salud* was scheduled for the weeks starting on 9 October (Mazo), 16 October (San Andrés y Sauces), 23 October (Los Llanos de Aridane), 6 November (El Paso), 13 November (Tazacorte), 20 November (Tijarafe), 11 December (Puntallana), and 2 January (Breña Baja). To control the participants’ attendance, a “passport” was designed and handed to each of the subjects in the first workshop. They were asked to bring their passport every day and told that they would enter a drawing for a healthy breakfast for two people if they took part in at least four of the five workshops. After finishing each of the *Compartiendo Salud* weeks, the participants were treated to a healthy breakfast to end the intervention.

During the development of the intervention, no adjustments had to be made to the protocol or the planned schedule.

### 2.4. Variables

The data collection and the unit of analysis were individual. The instrument to collect and measure the variables included in the study was a hetero-applied questionnaire with the following variables:

#### 2.4.1. Main Variable

- Feeling of unwanted loneliness. This was measured using the “UCLA (University of California at Los Angeles) Loneliness Scale” test validated in Spain. This instrument is based on three dimensions: relational connection, social connection, and self-perceived isolation. It uses a 4-point scale ranging from “never” to “frequently.” The 10-item version (a shortened version of the original 20-item version) was used, which has been translated to, adapted to, and validated for Spanish. Scores above 30 correspond to “no loneliness,” between 20 and 30 mean “moderate loneliness,” and less than 20 points is “severe loneliness” [29].

#### 2.4.2. Secondary Variables

- Social risk. This was measured using the Gijón Social Risk Detection questionnaire. It assesses family and economic situations, housing, social relations, and support from social networks. It uses a 5-point scale ranging from best to worse situation for responses to 5 items. Scores are as follows: good/acceptable social situation: from 5 to 9 points, social risk: from 10 to 14 points, and social problem: more than 15 points [30].
- Perceived social support. This was measured with Duke’s questionnaire. It assesses perceived social support and consists of 11 items, each one assessed with a Likert scale from 1 to 5. Scores equal to or higher than 32 indicate “normal support,” whereas less than 32 points means “low perceived social support” [31].
- Anxiety and depression. These were assessed using Goldberg’s questionnaire with two subscales: Anxiety and Depression. Each of them is structured with 4 initial screening items to determine the probability of a mental disorder and a second group with 5 items that are only formulated if positive answers are given to the screening questions (at least 2 in the Anxiety subscale and at least 1 in the Depression subscale). The type of response of the questionnaire is dichotomous (Yes/No). The cut-off points are as follows: scores equal to or higher than 4 for the Anxiety subscale, and values equal to or higher than 2 for Depression. In the geriatric population, it has been proposed to use it as a single scale, with a cut-off point equal to or higher than 6 [32].
- Social support (Yes/No). This included belonging to an organized group, being integrated in the area where they live, having a support network, participating in activities in free and/or leisure time, having a caregiver, and having support people.
- Defining characteristics for risk of loneliness and for social isolation (Yes/No). These included affective deprivation, emotional deprivation, physical isolation, social isolation, low social activity levels, change in physical aspect, explicit dissatisfaction with social connections, social reclusion, social behavior inconsistent with cultural norms, reporting feeling insecure in public, and reduced eye contact.
- Variables related to lifestyle habits. These included eating habits, physical activity, smoking, alcohol consumption, and sleep (sleep problem; restful sleep; naps).

#### 2.4.3. Sociodemographic Variables

- These included gender, marital status, age, housing arrangement: unipersonal, home-based care, social assistance, architectonic barriers, and alteration in any sensory capability.

The following variables were added to the ones in the reassessment (in addition to collecting observations for each of them): applying the knowledge acquired; distinguishing between eating habits, physical exercise, digital literacy, memory/music therapy, and sleep hygiene; the plate method; sugar consumption reduction; reading products’ labels; continued physical exercise; digital literacy application; correction of/improvement in sleep problems; new belonging to a municipal association or group; and satisfaction with the activity.

### 2.5. Data Collection Procedure

The interventions carried out in our project are not classified as medical interventions, but as educational and community health activities. Therefore, the study does not align with the clinical trial criteria defined by the International Committee of Medical Journal Editors (ICMJE) regarding design and scope, nature of the intervention, or timing of implementation. The study was conducted in compliance with current European and Spanish laws and regulations regarding personal data processing, communication, and transfer, adhering to the provisions set forth in Regulation (EU) 2016/679 of the European Parliament and Council, dated 27 April 2016 (GDPR), and in Spanish Organic Law 3/2018 on Personal Data Protection and Guarantee of Digital Rights, dated 5 December. The study was approved by the La Palma Health Services Management Office and by the provincial ethics committee on 27 July 2023.

Prior to data collection, an appointment was scheduled with the IT and Security coordinator at the La Palma Health Area to ensure compliance with the current regulation regarding the treatment of sensitive data. The participants were anonymized with an alphanumeric code, thus ensuring data confidentiality and safety as per the current regulation. The database was hosted on one of the institution’s servers, which can only be accessed by the lead researcher and her collaborators. The contents included in this database will remain on this device for the next 5 years, with a security mechanism that will request a username and password to access the data.

### 2.6. Data Analysis

The sample was described with the frequency of categories for the nominal and ordinal variables and with the mean–standard deviation or median–percentiles for the scalar ones, according to their distribution. The percentages of qualitative variables were calculated. In the bivariate analysis, Pearson’s Chi-square techniques were used to study associations.

To calculate the effect size, Cohen’s d with Hedges’ correction and the r coefficient [r = z/√N] were employed, using the Wilcoxon test z-value to compare and analyze the pre- and post-intervention differences based on the variables stated in the objectives; this was done with the McNemar test, Spearman’s Rho correlation, or Wilcoxon’s test, as appropriate. All the bilateral tests were performed at alpha significance levels <0.05, with the aid of SPSS 28.0 (IBM Corp. Released 2017. IBM SPSS Statistics, Version 28.0. Armonk, NY, USA: IBM Corp.).

## 3. Results

### 3.1. Description of the Sample

The intervention was initiated with 90 participants in eight of the nine Basic Health Districts from the La Palma Health Area. Six of them were excluded (four for only attending one workshop and two for not providing their informed consent to take part in the study).

Most of the participants were women (85.7%), with a mean age of 75.7 years (median: 75; standard deviation: 6.34; minimum: 65–minimum: 90). A total of eight groups were assembled, with a mean of 10.5 participants per group. The most numerous groups were those in the Tazacorte (n = 18) and Mazo (n = 16) Basic Health Districts.

The distribution by inclusion criteria was as follows: 52.4% lived alone, 48.8% presented the risk of loneliness Nursing Diagnosis label, and 22.6% were classified with the social isolation label. More than half of the participants under social risk (57.5%) or socially isolated (57.9%) did not live alone. No significant differences were observed regarding gender or inclusion criterion. The distribution by Basic Health Districts and criteria to be included in the study was not homogeneous. The high prevalence of the risk of loneliness in the El Paso (80%) and Las Breñas (77.8%) Basic Health Districts stands out, as well as that of social isolation in Puntagorda (62.5%).

In the participants diagnosed with risk of loneliness, the risk factors recorded were physical isolation (75%), social isolation (73.3%), affective deprivation (71.4%), and emotional deprivation (70.6%).

### 3.2. Pre-Intervention Analysis

Prior to the intervention, 32.1% of participants reported experiencing moderate levels of unwanted loneliness, while 12.3% were classified within the severe range according to the UCLA Loneliness Scale. The mean score was 30.25 (standard deviation: 7.85; median: 32).

Most of the participants had adequate eating habits (57%), were active (40,5%) or partially active (48.8%), and did not smoke (71.4%) or drink alcoholic beverages (77.4%). However, more than half of them mentioned having sleep problems (53.6%), although 66.7% stated that they enjoy restful sleep. Three out of 10 (32%) took naps for slightly more than one hour (1.09 h).

As for living conditions, 69% stated that they face architectonic barriers in their home, whether internal (13.1%), external (25%), or of both types (31%). In all, 11.9% of the subjects were included in home-based care programs that their Primary Care team was in charge of, and 15.5% were beneficiaries of some kind of social assistance.

In terms of social support, 73.8% indicated having people they could rely on, and 88.1% felt integrated within their local community. However, 42% reported not having a support network. Less than half of the subjects belonged to an organized group (48.8%) and only 10.7% stated that they have a caregiver. A total of 58.3% performed some activity in their free time, whereas 51.2% indicated low social activity levels and 40.5% claimed to be dissatisfied with their social connections.

The Gijón test classified 60.2% of the participants as at intermediate social risk, 28% as at normal or low social risk, and 2.4% as suffering an established social problem. It is noteworthy that 16.9% of the participants were living alone and had no children or, if they did, they lived away from them.

According to Goldberg’s Anxiety–Depression scale, 44% and 42.9% suffered depression and anxiety, respectively. More than 5 out of 10 participants were above the anxiety or depression thresholds (52.4%). No significant differences were evidenced between men and women.

Measured with the DUKE-UNC scale, perceived social support was recorded before the intervention in 95.2% of the participants. A total of 25.3% of them scored below 32 points, which implies low perceived social support. There were no significant differences by gender.

The score distribution corresponding to anxiety, depression, social risk, perceived social support, and feelings of unwanted loneliness was assessed with the Kolmogorov–Smirnov test, obtaining <0.05 in all cases (poor adjustment to normal distribution). When correlating the scores of these four scales, those that were significantly related when performing Spearman’s Rho test were the Anxiety and Depression subscales (0.553; *p* < 0.001), which indicates a moderate correlation.

No significant differences were observed between the degree of feelings of unwanted loneliness and gender, perceived social support, anxiety, depression, sleep, support network and people, adequate eating habits, physical activity, or architectonic barriers.

### 3.3. Post-Intervention Analysis

Three months after the intervention, 85% of the participants did not present feelings of unwanted loneliness, whereas 15% stated moderate levels, and no patients indicated a severe level. This indicates a 28.8% reduction (*p* < 0.001).

The mean score was 35.64 (standard deviation: 4.88; median: 37). The mean value of the difference between the scores obtained in this scale before and after the intervention was −5.39 (median: −4; standard deviation: 7). As can be seen in Table 2, a moderate effect size was evidenced (d = −0.77; 95%CI [−1.02, −0.52]), which implies that the intervention exerted a relevant impact, close to a large effect, according to the parameters proposed by Cohen (small: 0.2; moderate: 0.5; large: 0.8).

The Wilcoxon test revealed statistically significant differences (*p* < 0.001) in the mean values for unwanted loneliness, depression, and anxiety between pre- and post-intervention measurements, thereby rejecting the null hypothesis (Table 2).

**Figure 1:**
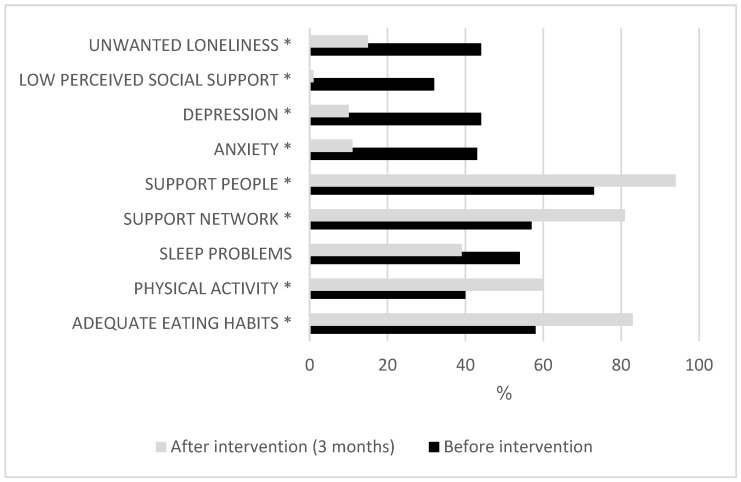
Differences before and after the intervention. * Statistically significant *p*-value. McNemar test to compare dichotomous qualitative variables.

The assessments of the participants three months after the proposed community-based intervention showed significant differences (*p* < 0.05) in adequate eating habits, physical activity, support network, anxiety, depression, perceived social support, and degree of feelings of unwanted loneliness (Figure 1, Table 3).

Measured with Goldberg’s scale, both anxiety and depression were reduced (*p* < 0.001). This happened in the Depression (9.5%) and Anxiety (10.8%) subscales alike. The mean value of the difference between the scores obtained in the Anxiety subscale before and after the intervention was 2 (median: 1; standard deviation: 3). A moderate effect size (d = 0.67; 95%CI [0.43, 0.90]) was evidenced. The mean value of the difference between the scores obtained in the Depression subscale before and after the intervention was 1.9 (median: 0; standard deviation: 2.9). A moderate effect size (d = 0.65; 95%CI [0.40, 0.89] was evidenced.

The score distribution for the Goldberg, Duke, and UCLA scales was assessed with the Kolmogorov–Smirnov test, obtaining <0.05 in all cases (poor adjustment to normal distribution). When correlating the scores obtained in these three scales, the significant correlations when performing Spearman’s Rho test were between anxiety and depression (0.516; *p* < 0.001) and between feelings of unwanted loneliness and perceived social support (0.429; *p* < 0.001), which implies a moderate correlation between these variables. This last relationship was not significantly present before the intervention.

The evolution corresponding to the questions from the Goldberg, Duke, and UCLA tests is shown in Table 4, Table 5 and Table 6. A significant reduction was noticed in each of the aspects assessed (*p* < 0.05), except for “being thirsty for company” (*p* = 0.023) and of “feeling silenced and excluded by other people” (*p* = 0.093).

Measured with the Duke–UNC scale, perceived social support shows that, three months after the community-based intervention, all but one patient perceived normal social support (*p* < 0.001), with a mean score of 45.05.

After the intervention, 83.3% of the participants maintained adequate eating habits. In turn, 79.5% stated having reduced their sugar consumption, 74.7% mentioned that they read the products’ labels, and 55.4% asserted to be using the plate method.

As for physical activity, 6 out of 10 participants considered themselves physically active. A total of 65% performed more physical activities than before the intervention and in a constant and sustained way (60%). A total of 75% stated having applied knowledge acquired in the workshops, especially in doing gymnastics (15%) and going out for a walk (18%).

Once the intervention had ended, 60% used some digital literacy tools, especially video calls (8.4%) and the “*Mi Cita Previa*” app from the SCS (6%).

In all, 66% of the participants performed activities related to memory and music therapy, with word search (12%) and memory games (12%) standing out.

The intervention contributed several improvements in terms of sleep, although they were not statistically significant (*p* = 0.36). Despite that, 45% of the participants mentioned having applied diverse knowledge acquired in the workshops in relation to sleep hygiene, especially relaxation (17%).

A total of 98% of the participants indicated that they would like for the activity to be sustained in time. The preferred periodicity was once a month (40%).

## 4. Discussion

*Compartiendo Salud* is a multicomponent intervention aimed at promoting the health of older adults that live alone, are at social risk, or are in social isolation. The impact exerted on the subjective feeling of unwanted loneliness and on health management is a relevant result of this research.

**Figure 2:**
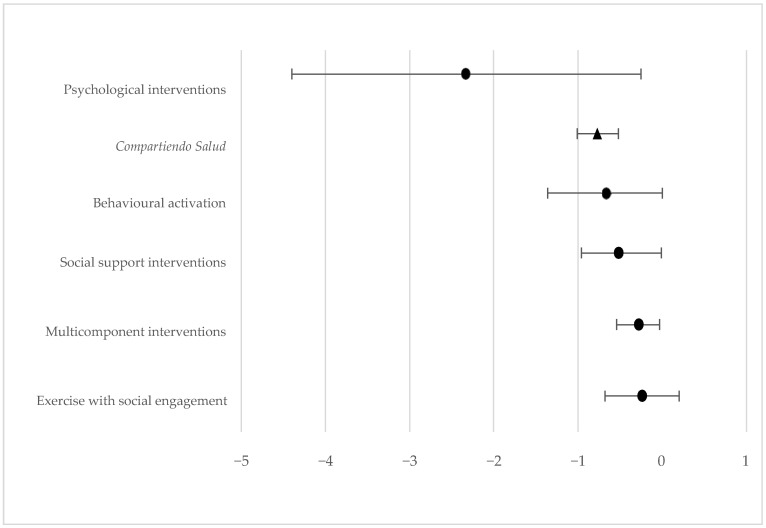
Descriptive forest plot. Adapted from the results obtained in the study by Saun-Fun Yu et al. [14], including those of *Compartiendo Salud*. Effect of the intervention proposed on unwanted loneliness. Cohen’s proposal is taken as a reference for the effect size as follows: 0.8—large; 0.5—moderate; 0.2—small. The design of the studies and the number of participants were not considered in the figure.

The proposed intervention produced a moderate effect on the subjective experience of unwanted loneliness in the study population. This is a promising result, especially considering that, in the specialized literature (except for psychological interventions, which show a strong effect), the other interventions yield a small or moderate effect on these individuals, as shown in Figure 2.

The study intervention not only exerted an impact on the feeling of unwanted loneliness in the aged population but also achieved significant improvements across each of the areas addressed during the intervention. Sleep quality improved, although not significantly. Given that insomnia and sleep problems are associated with the frail aged population, more studies addressing sleep disorders in this segment and their pathophysiological basis are required [33].

Levels of physical activity among older adults increased and were maintained three months following the intervention. In this improvement, the positive effect of the workshops’ dynamics should be considered, where the importance of the activity and the interview–assessment used (Nursing Diagnosis, counseling, and the approach of why it is necessary for them to change their habits) are explained [34]. The interventions to promote physical activity in PHC showed a small–moderate positive effect on the increase in physical activity levels [35]. The association between physical activity and healthy aging as positive was evident; however, so that comparisons with previous studies can be established, it is necessary to reduce heterogeneity in the definition of physical activity and in the way it is measured [36].

Living alone is directly associated with a deterioration in eating habits, often resulting from neglect in meal selection and dietary monotony [37]. Eating habits are one of the factors that exert an influence on the onset of dependence and the development of disabilities. Throughout the years, sensory alterations and mechanical difficulties when it comes to eating can cause problems with implementing a healthy diet. In *Compartiendo Salud*, emphasis is made on improving the quality of the participants’ eating habits, achieving the purpose of reducing sugar consumption and properly reading the products’ labels.

Anxiety is the most frequently recorded mental health problem in Primary Care medical records in Spain. This is an ailment with a relatively stable frequency between the ages of 35 and 84 years old [38]. In the study sample, 4 out of 10 participants had anxiety before the intervention, which was significantly reduced after three months (11%) but is still above the 6.7% of the Spanish population with a health card affected by this condition.

The prevalence of depression in our sample was high (44%). The Spanish National Health System estimates that depressive disorder is present in 4.1% of the population (5.9% in women and 2.3% in men) [39], progressively increasing to 12% in women and 5% of men aged 75 to 84 years old. However, after the intervention, the prevalence in our population aligned more closely with these reference figures (9.5%). Loneliness is strongly associated with depression and may in fact be an independent risk factor for depression.

The literature indicates that social support has a greater impact on reducing loneliness in rural areas compared to in urban settings, a difference that has not been validated in this study. The negative association between social support and loneliness is stronger among rural populations than among urban populations [40].

In our research, neither age nor presence of architectonic barriers was significantly associated with moderate or severe loneliness; however, it should be considered that our study excludes confined people, which can be an important bias when comparing both studies. A Catalan study divided the population that presents the feeling of unwanted loneliness into two groups: one that relates it to aging and to architectonic barriers, and another group of individuals that felt lonely despite living with other people [36]. The following profile was detected in our study: people that stated feeling lonely and the need to be heard and refusing invitations to take part in the available resources to join associations or activities, claiming to be satisfied with their current social activities, something that should be explored more in future research studies.

After the intervention, there was an impact on the feeling of perceived social support, obtaining a normal perception result in almost 50% of the population. Offering them a meeting and socialization place had an impact on perceived social support. This problem is so present nowadays that recording the social sphere and asking the participants about it should be incorporated into the different health teams’ consultation routine. In addition, Primary Care should provide the social and citizenship resources of its community to promote socialization and meeting activities and implement social prescription. Likewise, specific screening programs or procedures for loneliness that ease the identification of high-risk individuals should be incorporated.

This study is not exempt from limitations. Extreme caution should be taken when interpreting the effect of the intervention and in comparisons to studies with more robust designs. Although this limits the generalizability of the results, this design was selected for reasons of feasibility and resource management. The accessible population can be considerably smaller than the one to be studied. In addition, a larger sample would allow for a small effect size to be detected. In future research, it should be considered that exclusion of individuals with uncorrected hearing loss may lead to missing patients at social risk, as this deficit is associated with greater social fragility [41]. While the decision not to include a control group was made for ethical and logistical reasons, it does carry limitations in the interpretation of the results. Although the results are promising, studies with no control group cannot guarantee that the changes evidenced are due to the intervention itself, to other interventions, or to uncontrolled factors; however, the preliminary impact of the intervention can in fact be assessed. In subsequent surveys, aspects such as selection randomization, the interaction within the selection, and the inclusion of a control group should be considered. In future studies, it may be interesting to address the feeling of self-perceived loneliness from a qualitative perspective. On the other hand, the population dispersion in the La Palma island and the absence of a fluid public transportation service have imposed difficulties in reaching the intervention locus.

## 5. Conclusions

Loneliness and social isolation are social determinants of health that significantly affect older adults, compromising their quality of life. Identifying these issues is important due to their impact on the well-being, life expectancy, and quality of life of our population. This study demonstrates that a multidimensional community-based intervention can significantly reduce unwanted loneliness while improving social support, anxiety, and depression in older adults living alone. These promising results underscore the potential for integrating this intervention into regional and national public health strategies designed to address social isolation. The results of this intervention could serve as a model for designing replicable strategies in other communities, improving older adults’ quality of life and levels of perceived social support. The intervention’s design, community-based nature, and integration into routine community nursing practice suggest high potential for replication in similar rural or urban contexts beyond La Palma. Future studies should explore its applicability in different healthcare settings and geographic areas. Future research should confirm these findings through randomized controlled trials and explore the scalability of this intervention across diverse healthcare contexts.

## Figures and Tables

**Table 1:** Concepts and dynamics corresponding to each of the five sessions that comprise the *Compartiendo Salud* intervention.

Concepts	Dynamics
Sleep hygiene	
Importance of resting What is sleep? Conditioning factors Naps Pharmacological and non-pharmacological treatments Sleep hygiene, sleep and eating habits Sleep and physical exercise Relaxation techniques	Presentation with the ball Brainstorming: “Is it important to sleep?” Videos explaining the “7Ds” method When is it best to eat this food? To wake up or to go to bed? True or false Jacobson’s progressive muscle relaxation technique
Healthy and sustainable eating habits	
Importance of eating habits The “5S” of eating habits Food groups What to buy? Label reading Harvard plate	Presentation with the ball Identifying hidden spices What to buy? How much sugar is in this product? We create our meal. “Re-cipe me”: turning an unhealthy recipe into a healthy one
Physical activity and exercise	
Benefits of physical activity Types of physical exercise Warming up Adapted balance, resistance, strengthening, and flexibility exercises Stretching	Presentation with the ball Warming up Circuit including balance, resistance, strength, and flexibility exercises Ring game Stretching
Memory and music therapy	
Long- and short-term memory exercises Auditory and audiovisual memory activities Calculation, attention, and language exercises	Presentation with the ball Recalling old-time games and sayings Recalling series of numbers and words Identifying and recalling the highest possible number of elements from an image for 30 s Matching a person’s name with their photograph (actors/actresses, presenters, singers, public figures) Double-card game; searching for the match Replicating a geometrical shape Calculation exercise Listing a group of words without repeating those already mentioned Filling in the lyrics of a song with missing words Drawing a sketch representing a movie or a song for the rest of the group to guess Drama is your cup of tea: using mime to try and represent the lyrics of a song for the others to identify which song it is Playing “Color Esperanza” (“The Color of Hope”) and “Madre Tierra” (“Mother Earth”) and representing their lyrics with body movements
Digital literacy	
Format adjustments Connecting to a Wi-Fi network Using instant messages Using video calls, searching on the Internet Recording events and appointments in a calendar Listening to music Emergency contact “Mi Cita Previa,” “Mi Historia Clínica,” and “TILP” apps; taking photographs; and recording video	Presentation with the ball Explaining the different sections of the contents, helping the participants do it with their cell phones We downloaded apps that ease their management, such as those available in the SCS and the ones they requested.

**Table 2:** Effect size in the scores corresponding to feelings of unwanted loneliness and to the Anxiety and Depression subscales.

Variable (Score) *	d	g	r	Mdn Mdn Before After		Rg Neg	Rg Pos	z	*p*
Loneliness	−0.77	−0.77	0.65	32	37	274	2,355	−5.8	<0.001 **
Anxiety	0.67	0.66	0.58	2	0	1,768	249	−5.2	<0.001 **
Depression	0.65	0.64	0.55	1	0	1,181	146	−4.9	<0.001 **

* d: Cohen’s d; g: Hedges’ g; r coefficient: r = z/√; Mdn: median; Rg: sum of ranges; Z: Wilcoxon test statistical value. ** Statistically significant *p*-value in the Wilcoxon test context.

**Table 3:** Differences before and after the intervention.

		% After			
Variable	% Before	No	Yes	T	*p* *
Adequate eating habits	No	6	36.1	42.2	0.001 **
	Yes	10.8	47	57.8	
	T	16.9	83.1	100	
Physical activity	No	30.1	30.1	60.2	0.005 **
	Yes	9.6	30.1	39.8	
	T	39.8	60.2	100	
Sleep problems	No	36.9	9.5	46.4	0.36
	Yes	23.8	29.8	53.6	
	T	60.7	39.3	100	
Support network	No	11	31.7	42.7	0.001 **
	Yes	8.5	48.8	57.3	
	T	19.5	80.5	100	
Support people	No	1.2	25.6	26.8	<0.001 **
	Yes	4.9	68.3	73.2	
	T	6.1	93.9	100	
Anxiety (Goldberg)	No	52.4	4.8	57.2	<0.001 **
	Yes	36.9	6	42.9	
	T	89.3	10.8	100	
Depression (Goldberg)	No	53.6	2.4	56	<0.001 **
	Yes	36.9	7.1	44	
	T	90.5	9.5	100	
Low perceived social support (Duke)	No	67.9	0	67.9	<0.001 **
	Yes	30.9	1.2	32.1	
	T	98.8	1.2	100	
Unwanted loneliness (UCLA mod/sev)	No	53.8	2.5	56.3	<0.001 **
	Yes	31.3	12.5	43.8	
	T	85	15	100	

* McNemar test to compare dichotomous qualitative variables. ** Statistically significant *p*-value.

**Table 4:** Evolution of the questions from Goldberg’s instrument before the intervention and three months later.

		% After			
Variable	% Before	No	Yes	T	*p* *
Have you felt extremely excited, nervous, or tense?	No	34.9	12.0	46.9	0.001 **
	Yes	37.3	15.7	53	
	T	72.2	27.7	100	
Have you been extremely worried about something?	No	36.1	10.8	46.9	0.002 **
	Yes	36.1	18.1	54.2	
	T	72.2	28.9	100	
Have you felt extremely irritable?	No	65.1	3.6	68.7	<0.001 **
	Yes	26.5	4.8	31.3	
	T	91.6	8.4	100	
Have you had difficulties relaxing?	No	57.8	6.0	63.8	<0.001 **
	Yes	31.3	4.8	36.1	
	T	89.2	10.8	100	
Have you felt you had little energy?	No	39.8	10.8	50.6	0.003 **
	Yes	33.7	15.7	49.4	
	T	73.5	26.5	100	
Have you lost interest in things?	No	63.9	3.6	67.5	<0.001 **
	Yes	27.7	4.8	32.5	
	T	91.6	8.4	100	
Have you lost self-confidence?	No	79.5	1.2	80.7	0.002 **
	Yes	15.7	3.6	19.3	
	T	95.2	4.8	100	
Have you felt despair, hopelessness?	No	71.1	1.2	72.3	<0.001 **
	Yes	26.5	1.2	27.7	
	T	97.6	2.4	100	

* McNemar test to compare dichotomous qualitative variables. ** Statistically significant *p*-value.

**Table 5:** Evolution of the questions from Duke’s instrument before the intervention and three months later.

		% After			
Variable	% Before	No	Yes	T	*p* *
I receive visits from my friends and relatives.	NO	22.9%	41.0%	63.9%	<0.001 **
	YES	8.4%	27.7%	36.1%	
	T	31.3%	68.7%	100.0%	
I have help with issues related to my house.	No	13.3%	47.0%	60.2%	<0.001 **
	Yes	6.0%	33.7%	39.8%	
	T	19.3%	80.7%	100.0%	
I receive compliments and acknowledgments when I do my job well.	No	6.0%	47.0%	53.0%	<0.001 **
	Yes	3.6%	43.4%	47.0%	
	T	9.6%	90.4%	100.0%	
I receive love and affection.	No	7.2%	22.9%	30.1%	<0.001 **
	Yes	2.4%	67.5%	69.9%	
	T	9.6%	90.4%	100.0%	
I can talk to someone about my problems at work or at home.	No	2.4%	33.7%	36.1%	<0.001 **
	Yes	6.0%	57.8%	63.9%	
	T	8.4%	91.6%	100.0%	
I can talk to someone about my personal and family problems.	No	3.6%	37.3%	41.0%	<0.001 **
	Yes	9.6%	49.4%	59.0%	
	T	13.3%	86.7%	100.0%	
I can talk to someone about my personal and economic problems.	No	4.8%	39.8%	44.6%	<0.001 **
	Yes	7.2%	48.2%	55.4%	
	T	12.0%	88.0%	100.0%	
I receive invitations to entertain myself and go out with other people.	No	8.4%	39.8%	48.2%	<0.001 **
	Yes	8.4%	43.4%	51.8%	
	T	16.9%	83.1%	100.0%	
I receive useful advice when there is some important event in my life.	No	1.2%	45.8%	47.0%	<0.001 **
	Yes	2.4%	50.6%	53.0%	
	T	3.6%	96.4%	100.0%	
I have help when I’m ill in bed.	No	4.8%	30.1%	34.9%	<0.001 **
	Yes	2.4%	62.7%	65.1%	
	T	7.2%	92.8%	100.0%	

* McNemar test to compare dichotomous qualitative variables. ** Statistically significant *p*-value. Grouping of answers: No (“Far less”/“Less than what I wish”/“Neither very much nor little”); Yes (“Almost as much”/“As much as I wish”). In the pre-intervention assessment, the “I have people that are worried about what happens to me” question was not recorded in some participants (but it was in fact considered in the test result), which is why it is excluded from this analysis.

**Table 6:** Evolution of the questions from the UCLA instrument before the intervention and three months later.

		% After			
Variable	% Before	O/F	R/N	T	*p* *
How often do you feel unhappy doing so many things by yourself?	O/F	9.6%	27.7%	37.3%	0.002 **
	R/N	7.2%	55.4%	62.7%	
	T	16.9%	83.1%	100.0%	
How often do you feel that you have no one to talk to?	O/F	6.0%	30.1%	36.1%	<0.001 **
	R/N	2.4%	61.4%	63.9%	
	T	8.4%	91.6%	100.0%	
How often do you feel that you can’t bear feeling alone?	O/F	6.0%	19.3%	25.3%	<0.001 **
	R/N	2.4%	72.3%	74.7%	
	T	8.4%	91.6%	100.0%	
How often do you feel that nobody understands you?	O/F	1.2%	25.3%	26.5%	<0.001 **
	R/N	2.4%	71.1%	73.5%	
	T	3.6%	96.4%	100.0%	
How often do you find yourself waiting for someone to call you or write to you?	O/F	3.6%	24.1%	27.7%	<0.001 **
	R/N	3.6%	68.7%	72.3%	
	T	7.2%	92.8%	100.0%	
How often do you feel completely alone?	O/F	7.2%	22.9%	30.1%	<0.001 **
	R/N	3.6%	66.3%	69.9%	
	T	10.8%	89.2%	100.0%	
How often do you feel incapable of reaching those around you and communicating with them?	O/F	6.0%	22.9%	28.9%	<0.001 **
	R/N	0%	71.1%	71.1%	
	T	6.0%	94.0%	100.0%	
How often do you feel thirsty for company?	O/F	8.4%	21.7%	30.1%	0.023 **
	R/N	7.2%	62.7%	69.9%	
	T	15.7%	84.3%	100.0%	
How often do you feel that you find it difficult to make friends?	O/F	8.4%	28.9%	37.3%	<0.001 **
	N	3.6%	59.0%	62.7%	
	T	12.0%	88.0%	100.0%	
How often do you feel silenced and excluded by other people?	O/F	1.2%	19.3%	20.5%	0.093
	R/N	8.4%	71.1%	79.5%	
	T	9.6%	90.4%	100.0%	

* McNemar test to compare dichotomous qualitative variables. ** Statistically significant *p*-value. R/N: rarely/never;—O/F: oftentimes/frequently.

## Data Availability

The original contributions presented in this study are included in the article. Further inquiries can be directed to the corresponding author.
